# The Biomechanical Characteristics of Swallowing in Tracheostomized Patients with Aspiration following Acquired Brain Injury: A Cross-Sectional Study

**DOI:** 10.3390/brainsci13010091

**Published:** 2023-01-03

**Authors:** Xiao-Xiao Han, Jia Qiao, Zhan-Ao Meng, Dong-Mei Pan, Ke Zhang, Xiao-Mei Wei, Zu-Lin Dou

**Affiliations:** 1 Department of Rehabilitation Medicine, the Third Affiliated Hospital of Sun Yat-sen University, No. 600, Tianhe Road, Tianhe District, Guangzhou 510630, China; 2 Department of Radiology, the Third Affiliated Hospital of Sun Yat-sen University, No. 600, Tianhe Road, Tianhe District, Guangzhou 510630, China; 3 Department of mechanical and automotive engineering, South China University of Technology, No. 381, Wushan Road, Tianhe District, Guangzhou 510641, China

**Keywords:** acquired brain injury, tracheostomy, aspiration, biomechanical characteristics, subglottic pressure, computational fluid dynamics

## Abstract

Objectives: Investigate the biomechanical characteristics in tracheostomized patients with aspiration following acquired brain injury (ABI) and further explore the relationship between the biomechanical characteristics and aspiration. Methods: This is a single-center cross-sectional study. The tracheostomized patients with aspiration following ABI and age-matched healthy controls were recruited. The biomechanical characteristics, including velopharynx (VP) maximal pressure, tongue base (TB) maximal pressure, upper esophageal sphincter (UES) residual pressure, UES relaxation duration, and subglottic pressure, were examined by high-resolution manometry and computational fluid dynamics simulation analysis. The penetration–aspiration scale (PAS) score was evaluated by a videofluoroscopic swallowing study. Results: Fifteen healthy subjects and fifteen tracheostomized patients with aspiration following ABI were included. The decreased VP maximal pressure, increased UES residual pressure, and shortened UES relaxation duration were found in the patient group compared with the control group (*p* < 0.05). Furthermore, the subglottic pressure significantly decreased in patients (*p* < 0.05), while no significant difference was found in TB maximal pressure between groups (*p* > 0.05). In addition, in the patient group, VP maximal pressure (*r*_s_ = −0.439; *p* = 0.015), UES relaxation duration (*r*_s_ = −0.532; *p* = 0.002), and the subglottic pressure (*r*_s_ = −0.775; *p* < 0.001) were negatively correlated with the PAS score, while UES residual pressure (*r*_s_ = 0.807; *p* < 0.001) was positively correlated with the PAS score (*p* < 0.05), the correlation between TB maximal pressure and PAS score (*r*_s_ = −0.315; *p* = 0.090) did not reach statistical significance. Conclusions: The biomechanical characteristics in tracheostomized patients with aspiration following ABI might manifest as decreased VP maximal pressure and subglottic pressure, increased UES residual pressure, and shortened UES relaxation duration, in which VP maximal pressure, UES relaxation duration, subglottic pressure, and UES residual pressure were correlated with aspiration.

## 1. Introduction

Aspiration is of major concern to patients with dysphagia, and refers to food or gastric contents getting into the respiratory tract from the laryngopharyngeal or stomach before, during, or after swallowing, which improves the risks of pneumonia, leads to malnutrition and dehydration over the long term, and increases mortality [1,2,3,4]. For tracheostomized patients following acquired brain injury (ABI), aspiration is a common sequela [5]. ABI is defined as brain injuries caused by strokes, trauma, cerebral anoxia, tumors, or infections, which heavily impact the patient’s quality of life and increase the economic burden [6]. The neurophysiological changes following ABI usually lead to aspiration [7]. In addition, tracheostomy is usually performed in patients requiring prolonged mechanical ventilation during the first days after the acute event, which causes decreases in the strength of sensory input and disuse atrophy of laryngeal structures [8]. It has been reported that the incidence of aspiration in tracheostomized patients was about 59% [9]. Tracheostomy after ABI is accompanied by a series of pathological changes in the biomechanics of the pharynx and the upper respiratory tract [10]. In addition, the reduction in glottic airflow after tracheostomy results in a corresponding inability to generate subglottic pressure during swallowing [11], which has been proven to be closely associated with swallowing function [12]. These factors may lead to aspiration in tracheostomized patients following ABI.

The pharyngeal contraction and UES relaxation are important components of the pharyngeal swallow, in which the velopharynx (VP) maximal pressure, UES residual pressure, and tongue base (TB) maximal pressure are important indexes that reflect the pharyngeal and UES function, these parameters may be used in prediction of aspiration [13]. The solid-state high-resolution manometry (HRM) and videofluoroscopic swallowing study (VFSS) are widely used to evaluate biomechanical characteristics and aspiration during swallowing in clinical practice [14]. However, little research regarding the assessment of the biomechanical characteristics in tracheostomized patients following ABI with the use of HRM has been reported yet.

Subglottic pressure is an important biomechanical parameter defined as the pressure measured when the vocal cords are adducted. It may be related to aspiration by participating in swallow–breathing coordination [15,16]; however, there is insufficient evidence to draw definite conclusions about the correlation between subglottic pressure and aspiration. Multiple methods have been applied in the subglottic pressure measurement. Studies attempted to use lung volume changes and intraoral air pressure during lip closure while making a “pa” sound with a pressure transducer to infer the subglottic pressure, but the reliability of these methods is controversial [16,17]. In addition, healthy volunteers underwent a direct measurement (cricothyroid puncture), an invasive procedure, to assess the subglottic pressure [18,19]. Therefore, an effective and noninvasive measurement method is needed for subglottic pressure assessment. Computational fluid dynamics (CFD) is a validated method to compute the airflow of the upper airway accurately and can be used to describe the pressure distribution of the upper airway, which provides a feasible method for the subglottic pressure measurement [20,21].

Currently, research using CFD to evaluate subglottic pressure is rare, and there is no consensus about the biomechanical characteristics in tracheostomized patients following ABI. In order to address this knowledge gap, we not only adopted CFD to compute the subglottic pressure according to a 3D reconstruction of the upper airway but used HRM to assess the biomechanical parameters, including VP maximal pressure, UES residual pressure, TB maximal pressure, and UES relaxation duration. The objective of this study was to investigate the biomechanical characteristics in tracheostomized patients with aspiration following ABI and further explore the relationship between the biomechanical characteristics and aspiration.

## 2. Methods

This is a single-center cross-sectional study, which was approved by the ethics committee of the Third Affiliated Hospital of Sun Yat-sen University (Ethics No. [2018] 02-374-01), and registered at the China Clinical Trial Center (Registration No. ChiCTR1800018686). All control subjects, patients, and their legal guardians had provided written informed consent.

### 2.1. Subjects

The tracheostomized patients with aspiration following ABI and age-matched healthy controls were recruited. For patients, the inclusion criteria were: (1) age ranging from 18 to 80 years; (2) with a tracheostomy tube; (3) not mechanically ventilated; (4) without requirements for supplementary oxygen; (5) manifesting as aspiration (penetration–aspiration scale (PAS) score ≥ 5). The exclusion criteria were: (1) unstable vital signs; (2) severe cognitive impairment; (3) a poor motor function that cannot cooperate with the inspection and evaluation; (4) previous diagnosis of dysphagia; (5) a history of seizure; (6) allergy to iohexol injections; (7) any neuropsychiatric comorbidity, affective disorder, or other respiratory diseases that may influence the test outcomes. For healthy participants, the inclusion criteria were: (1) age range from 18 to 80 years. The exclusion criteria were: (1) unstable vital signs; (2) previous diagnosis of dysphagia; (3) a history of seizure; (4) allergy to iohexol injections.

The Cuffed Blue Line Ultra^®^ Suctionaid^®^ tracheostomy tube (Smiths Medical ASD, Inc., Minneapolis, MI, USA) was used in the study, which is made of silicone with a soft seal cuff, and the size ranged from 7.0 to 8.5 mm. Cuffs were inflated (the pressure ranged from 20 to 30 cmH_2_O) during the evaluation.

The baseline information includes age, gender, body mass index (BMI), Barthel index (BI) score, functional oral intake scale (FOIS) score, and pulmonary infection. Brain injury etiology, lesion location, lesion side, NIHSS (National Institute of Health Stroke Scale for stroke), time of ABI onset, and duration of tracheal intubation were investigated in the patient group.

### 2.2. HRM Procedure

The biomechanical characteristics were detected by the HRM system (Sierra Scientific Instruments, Los Angeles, CA, USA). The major parameters, including VP maximal pressure, TB maximal pressure, UES residual pressure, and UES relaxation duration, were investigated. VP was defined as the zone of swallow-related pressure change proximal to the region of continuous nasal nostril quiescence extending 2 cm distally. TB was defined as the zone of swallow-related pressure change, with a high-pressure area midway between the VP and UES, with its center at the maximal pressure point and extending 2 cm proximal and distal to that point. The UES region was defined as the midpoint of stable high pressure just distal to the baseline low esophageal pressure zone [22,23]. Two investigators worked together in the data acquisition; if inconsistencies in the records happened, the measures were double-checked and proofread.

The device used the proprietary pressure transduction technology (TactArray), which allowed each of the 36 pressure-sensing elements to detect pressure over a length of 2.5 mm in each of the 12 circumferentially dispersed sectors. The sector pressures were then averaged to obtain the mean pressure, making each of the 36 sensors a circumferential pressure detector. The change in the electrical signal on the sensor directly reflected the pressure change, and the pressure measurements were expressed in terms of atmospheric pressure [23].

Oral cleaning, sputum suction, and nasogastric feeding tube extraction were performed before the examination. All subjects were required to be in a natural sitting position with their head in a neutral position, then underwent transnasal placement of the manometric catheter, and the catheter was fixed in place by taping it to the nostril. After a 10 min adaptation period, the subject was instructed to swallow 5 mL iohexol injection for a total of three swallows, and the data were analyzed using ManoView analysis software (Sierra Scientific Instruments, Los Angeles, CA, USA) [22,23].

### 2.3. VFSS Procedure

The subjects were placed in a neutral sitting position under the guidance of a C-arm remote control twin-perspective gastrointestinal X-ray machine (Toshiba DBA-300, Toshiba Ltd. Co, Tokyo, Japan). Each subject was instructed to swallow 5 mL iohexol injection for a total of three swallows. The severity of airway invasion during swallowing was measured by the PAS score, and the average value of the PAS score was included in the final analysis. The PAS is a valid, ordinal scale that evaluates depth, patient response, and clearance of airway invasion. Scores range from 1 (no penetration or aspiration) to 8 (silent aspiration) [24].

### 2.4. Reconstruction of the Upper Airway Model

The CT scanner (Aquilion ONE; Toshiba Medical Systems Corp., Tokyo, Japan) was used for the 3D model construction of the upper airway. Subjects were placed in the supine position during the CT examination. The full sequence was performed in approximately 8.9 s, and the tube voltage/current was 120 kV/60 mA. The coronal, sagittal, and axial planes CT images of the upper airway with a 0.5 mm thickness were obtained. The dataset was analyzed using Mimics software (version 20.0, The Materialise Group, Leuven, Belgium) to construct the 3D model. In order to keep the patient-specific characteristics of the upper airway shape, an appropriate smoothing algorithm was used to transform the 3D model into a smooth model. Subsequently, stereolithography files of the 3D models were imported into ANSYS 15.0-Meshing (version 15.0, ANSYS, Canonsburg, PA, USA) for model modification and mesh generation.

### 2.5. CFD Procedure to Assess the Subglottic Pressure

At first, the mesh of the 3D geometry model was generated with the unstructured meshes to capture the details of the upper airway. The quality and accuracy of the mesh were checked to satisfy the requirements. After mesh generation, the mesh file was imported into ANSYS FLUENT 15.0 to carry out simulations to obtain the velocity and pressure distribution in the upper airway.

During the simulation process, the upper airway was regarded as a rigid cavity, and the fluid flowed in an incompressible manner with constant viscosity. Two different phases, respiratory and swallowing, were considered in the simulation, so the boundary conditions were determined under the two phases. During the respiratory phase, the respiratory cycle duration was T = 3 s. The outlet of the subglottic cavity was defined as the velocity outlet, the value of which is defined as the respiration volume. The pharyngeal pressure was used as the pressure value measured by HRM. During the swallowing phase, the duration of swallowing was defined as the time between the onset of VP contraction and post-deglutitive UES pressure peak [25,26]. The pressure inlet was defined as the boundary condition for palatopharyngeal entrance. The outlet of the subglottic cavity is at the pressure exit. The k-є standard Reynolds model was used to evaluate the turbulent flow within the upper respiratory tract. The mean subglottic pressure during swallowing was used for the final analysis.

### 2.6. Statistical Analysis

Continuous variables were presented as mean ± standard deviation (SD), and categorical variables as frequencies. The homogeneity of variance was measured using Levene’s test. Normally distributed data were determined by using the Shapiro–Wilk test. Fisher’s exact test was used to analyze the gender between groups. The two-independent sample *t*-test or Wilcoxon signed-rank test was used in clinical characteristics including age, BMI, BI, FOIS, and biomechanical characteristics, including VP maximal pressure, TB maximal pressure, UES residual pressure, UES relaxation duration, and subglottic pressure between groups. Spearman’s correlation analysis was performed to evaluate the correlation between VP maximal pressure, TB maximal pressure, UES residual pressure, UES relaxation duration, subglottic pressure, and PAS score. The level of statistical significance was set at *p* < 0.05. All statistical analyses were conducted using SPSS software (version 20.0, IBM, New York, NY, USA), and statistical charts were accomplished with the use of GraphPad Prism (version 9.0, Graphpad Software Inc., San Diego, CA, USA).

## 3. Results

### 3.1. Clinical Characteristics

**Figure 1:**
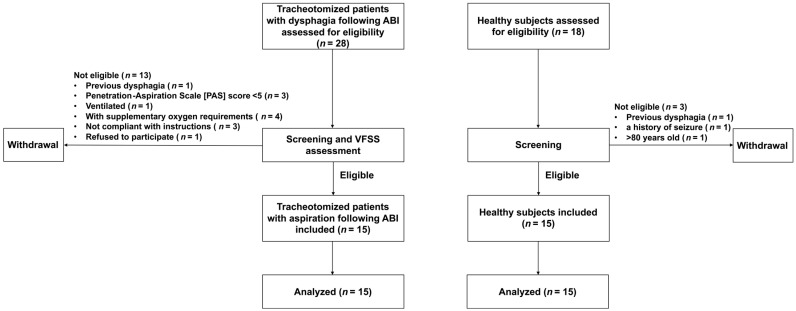
Participants’ flow diagram.

Fifteen tracheostomized patients with aspiration following ABI and fifteen healthy participants were enrolled in this study. The recruitment flow diagram is shown in Figure 1. The clinical characteristics of the study population are shown in Table 1. No significant differences were found in age and gender between the groups (*p* = 0.595, *p* = 0.462, respectively), while tracheostomized patients with aspiration following ABI presented with significantly lower BMI (*p* = 0.026), lower BI score (*p* < 0.001), and lower FOIS score (*p* < 0.001) as compared to healthy participants. Furthermore, the incidence of aspiration pneumonia in patients was 86.7%.

### 3.2. Comparison of the Biomechanics Characteristics during Swallowing between Groups

**Figure 2:**
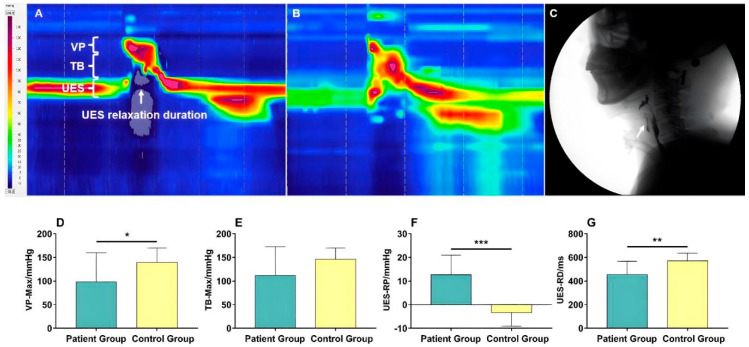
Comparison of the biomechanics characteristics between the patient group and control group: (**A**) Time–space chart of HRM in a healthy participant (white arrow, the UES relaxation duration). (**B**) Time–space chart of HRM in a tracheostomized patient with aspiration following ABI. (**C**) Aspiration occurred during swallowing in tracheostomized patients following ABI during VFSS examination (white arrow, the bolus entering the upper airway). (**D**–**G**) Comparison of VP–Max, TB–Max, UES–RP, and UES–RD between patients and control groups. There was a significant difference in VP–Max (**D**, *p* < 0.05), UES–RP (**F**, *p* < 0.001), and UES–RD (**G**, *p* < 0.01) between groups. Note: VP–Max, velopharynx maximal pressure; TB–Max, tongue base maximal pressure; UES–RP, UES residual pressure; UES–RD, UES relaxation duration. * *p* < 0.05; ** *p* < 0.01; *** *p* < 0.001.

The biomechanical parameters, including VP maximal pressure, TB maximal pressure, UES residual pressure, and UES relaxation duration during swallowing, were measured using HRM (Figure 2A,B), and the PAS score was assessed by VFSS (Figure 2C). Compared with healthy subjects, the results showed that tracheostomized patients with aspiration following ABI had significantly decreased VP maximal pressure (98.26 ± 61.57 vs. 139.41 ± 30.62 mmHg) (*p* = 0.031) (Figure 2D), significantly increased UES residual pressure (12.66 ± 8.29 vs. −3.31 ± 5.80 mmHg, *p* < 0.001) (Figure 2F), and shortened UES relaxation duration (454.40 ± 112.48 vs. 571.07 ± 65.56 ms, *p* = 0.002) (Figure 2G). However, the TB maximal pressure (111.83 ± 60.90 vs. 146.11 ± 24.02 mmHg) (*p* = 0.057) did not reach statistical significance (Figure 2E).

**Figure 3:**
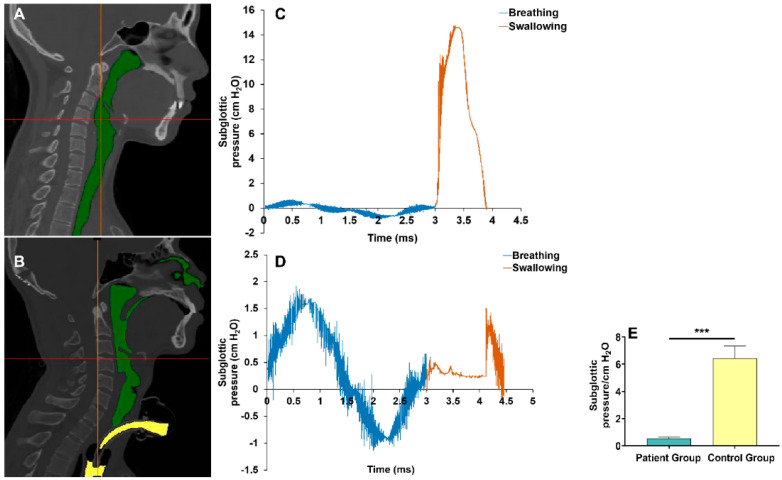
Comparison of the subglottic pressure during swallowing between the patient group and control group: (**A**) Three–dimensional reconstruction of the upper airway anatomical structure in a healthy subject (green area, upper respiratory tract). (**B**) Three–dimensional reconstruction of the upper airway anatomical structure in a tracheostomized patient with aspiration following ABI (green area, upper respiratory tract; yellow area, tracheal tube). (**C**) The trend of subglottic pressure in a healthy subject during swallowing. (**D**) The trend of subglottic pressure in a tracheostomized patient with aspiration following ABI during swallowing. (**E**) Comparison of the subglottic pressure during swallowing showed there was a significant difference between the patient group and control group. Note: *** *p* < 0.001.

A 3D upper airway anatomical reconstruction was made based on CT scans (Figure 3A,B). Subsequently, the subglottic pressure was analyzed using CFD (Figure 3C,D). Results showed that subglottic pressure ranged from 0.41 to 0.68 cmH_2_O in tracheostomized patients with aspiration following ABI and 5.26 to 8.39 cmH_2_O in healthy subjects. Compared with healthy subjects, tracheostomized patients with aspiration following ABI had significantly decreased subglottic pressure (0.54 ± 0.08 vs. 6.45 ± 0.89 cmH_2_O, *p* < 0.001) (Figure 3E).

### 3.3. Correlation between VP Maximal Pressure, TB Maximal Pressure, UES Residual Pressure, UES Relaxation Duration, Subglottic Pressure, and PAS Score in the Patient Group

**Figure 4:**
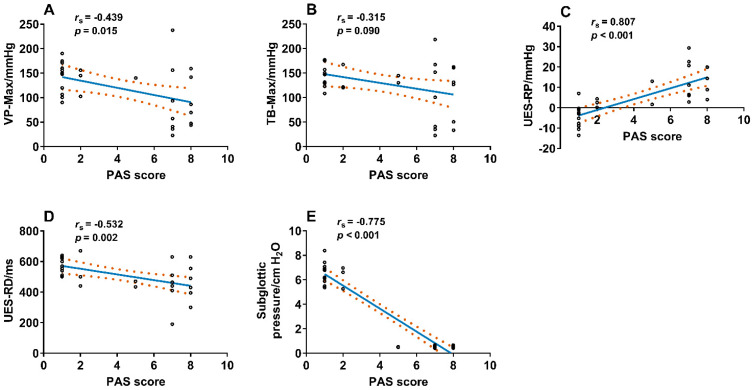
Correlation between VP–Max, TB–Max, UES–RP, UES–RD, subglottic pressure, and PAS score in the patient group. VP–Max (**A**), *r_s_* = −0.439, *p* = 0.015, UES–RD (**D**), *r_s_* = −0.532; *p* = 0.002, and subglottic pressure (**E**), *r_s_* = −0.775; *p* < 0.001 were negatively correlated with PAS score; UES–RP (**C**), *r_s_ =* 0.807, *p* < 0.001 was positively correlated with PAS score, and the correlation between TB–Max and PAS score was not significant (**B**), *r_s_* = −0.315, *p* = 0.090. Note: VP–Max, velopharynx maximal pressure; TB–Max, tongue base maximal pressure; UES–RP, UES residual pressure; UES–RD, UES relaxation duration; PAS score, penetration–aspiration scale score.

The Spearman’s correlation analysis showed that VP maximal pressure (*r_s_* = −0.439; *p* = 0.015), UES relaxation duration (*r_s_* = −0.532; *p* = 0.002), and the subglottic pressure (*r_s_* = −0.775; *p* < 0.001) were negatively correlated with the PAS score, UES residual pressure (*r_s_* = 0.807; *p* < 0.001) was positively correlated with the PAS score. However, the correlation between TB-Max and PAS score (*r_s_* = −0.315; *p* = 0.090) did not reach statistical significance (Figure 4).

## 4. Discussion

The major findings of the present study were as follows: (i) compared with healthy subjects, the tracheostomized patients with aspiration following ABI had significantly decreased VP maximal pressure, increased UES residual pressure, shortened UES relaxation duration, and decreased subglottic pressure; (ii) the VP maximal pressure, UES residual pressure, UES relaxation duration, and subglottic pressure were correlated with the PAS score.

The VP closure can not only prevent nasal reflux but form a sealed cavity between the soft palate and pharyngeal wall when the pharynx contracts, which is the first power source in the process of accumulating pressure during swallowing [27,28]. The present study demonstrated that the VP maximal pressure of tracheostomized patients with aspiration following ABI significantly decreased compared with that of healthy subjects, and VP maximal pressure was negatively correlated with the PAS score. The reasons why decreased VP maximal pressure was found in the patient group may be as follows: First, tracheostomized patients with dysphagia following ABI usually request long-term nasogastric feeding may lead to laryngeal musculature disuse. Second, the ABI may damage the neuroregulation of the levator veli palatini muscle, which can lead to VP insufficiency [29]. Third, neuropathy in critically ill, including ABI patients, may suffer from critical illness polyneuropathy (CIP) and myopathy (CIM), which is a potential reason for damaged swallowing-related cranial nerves and muscles [30,31]. Lastly, research indicated that ABI-induced neuroinflammation and neurodegeneration impact gut function, which can lead to hypermetabolism, hypercatabolism, and intolerance to enteral nutrition, resulting in quick depletion of nutrition reserves [32,33]; therefore, the VP maximal pressure was affected. VP insufficiency can lead to nasal reflux and pharynx pressure reduction, often resulting in the food bolus being transported into the esophagus inefficiently and a lack of clearance during swallowing [27], which may be an important factor in the occurrence of aspiration. Another study also indicated that decreased VP maximal pressure was an important predictor of aspiration pneumonia [34].

The present study failed to find a statistical difference in TB maximal pressure between patients and healthy subjects, and the correlation between TB-Max and PAS score did not reach statistical significance. The elevation of the hyoid–laryngeal complex may affect the pressure measurement in the nearby TB sensors and disturb the precise TB pressure measurement during HRM examination [35]. In addition, TB movement is modulated by volitional control, which may be influenced by patient cooperation [34]. Therefore, the measurement of TB pressure by HRM may be insensitive and potentially susceptible to interference, and VP maximal pressure may be more sensitive and representative than TB pressure in HRM examination.

Multiple factors influence the UES opening, in which the cholinergic contractions of UES, as well as pressure in the pharynx and esophagus, may become involved [36,37]. The UES residual pressure and UES relaxation duration reflect the status of UES during swallowing. The present study found that the tracheostomized patients with aspiration following ABI exhibited a higher UES residual pressure and shorter UES relaxation duration, indicating that an incomplete UES opening in such patients, which can cause food bolus transport into the esophagus inefficiently. Therefore, the pharyngeal residual might increase under these circumstances, which may increase the risk of aspiration during swallowing; this could explain why UES relaxation duration was negatively correlated with the PAS score and why UES residual pressure was positively correlated with the PAS score in the present study. Previous studies found that the UES relaxation duration was shorter in aspiration patients as compared to those who were not, and UES relaxation duration was associated with the development of aspiration pneumonia, which supported the validity of our original work [34,38].

The 3D reconstruction of the upper airway anatomical structure combined with CFD analysis was used to assess the subglottic pressure, and the pharyngeal pressure measured by HRM was defined as the boundary condition for the CFD analysis in the present study. The results showed that subglottic pressure ranged from 5.26 to 8.39 cmH_2_O in healthy subjects, which is consistent with the pressure measured by percutaneous puncture of the cricothyroid membrane (5.5 to 9.5 cmH_2_O, and 7 to 11 cmH_2_O, respectively) in two previous research [18,19]. On the other hand, the present study showed that subglottic pressure ranged from 0.41 to 0.68 cmH_2_O in tracheostomized patients with aspiration following ABI, which was significantly lower than in the healthy subjects. A previous study reported that the subglottic pressure was 0 cmH_2_O in tracheostomy patients, which is similar to our results [11]. Therefore, the subglottic pressure was detected with relatively high accuracy in our study. We found that the subglottic pressure was correlated with the PAS score. The subglottic pressure receptors in the larynx participate in the regulation of subglottic pressure. Tracheal air pressure may stimulate subglottic receptors that trigger a specific segmental swallowing reflex in the brain stem. When subglottic pressure is eliminated, it could hinder the drive of subglottic receptors, which may result in aspiration [10,11,12].

Given that those patients manifest decreased VP maximal pressure, increased UES residual pressure, shortened UES relaxation duration, and decreased subglottic pressure during swallowing, suitable preventive and treatment measures may be considered for tracheostomized patients with aspiration following ABI based on our results. First, swallowing exercises, including supraglottic swallow, super-supraglottic swallow, and effortful swallow with and without breath-hold, may be beneficial to the subglottic pressure and VP maximal pressure reconstruction [39]. Second, the Passy–Muir valve, which was reported in our previous study, is suitable for these patients to reconstruct the pressure gradient of the upper airway [40].

There are several limitations in the present study that need to be addressed. First, the change in biomechanics characteristics and the occurrence of aspiration in tracheostomized patients following ABI are mainly attributed to the tracheostomy or damaged central nervous after ABI remain unclear. Second, this is a small-sample-size and single-center study, which may be subject to a patient-selection bias. Third, the tongue is an important applicator of force to drive a bolus through the oral to pharyngeal, and lingual pressure generation plays a crucial role in oropharyngeal swallowing [41,42]. However, this study did not involve the measurement of lingual pressure. Therefore, the analysis and interpretation of the results should be considered cautiously, and such results need to be replicated in a larger sample.

## 5. Conclusions

In conclusion, the tracheostomized patients with aspiration following ABI might manifest as decreased VP maximal pressure, increased UES residual pressure, shortened UES relaxation duration, and decreased subglottic pressure during swallowing. Moreover, the VP maximal pressure, UES relaxation duration, and subglottic pressure were negatively correlated with the PAS score, and UES residual pressure was positively correlated with aspiration in such patients. Suitable preventive measures, including swallowing exercises, Passy–Muir valve, and nutritional support, may be considered in tracheostomized patients with aspiration following ABI to reduce the risk of aspiration in clinical practice.

## Figures and Tables

**Table 1:** Clinical characteristics in the patient and control groups.

	Patient Group (*n* = 15)	Control Group (*n* = 15)	*p* Value
Age (years), mean ± SD	59.21 ± 10.53	57.32 ± 9.65	0.595
Gender (male), *n* (%)	7 (46.7)	10 (66.7)	0.462
BMI (kg/m^2^), mean ± SD	18.97 ± 1.51	20.28 ± 1.65	0.026
BI score, mean ± SD	29.00 ± 15.72	98.00 ± 3.68	<0.001
FOIS score, *n* (%)			<0.001
1–3	15 (100)	0	
4–5	0	0	
6–7	0	15 (100)	
Brain injury etiology, *n* (%)			
Stroke	11 (73.3)	-	-
Brain tumor	2 (13.3)	-	-
Traumatic brain injury	2 (13.3)	-	-
Lesion location, *n* (%)			
Supratentorial	4 (26.7)	-	-
Infratentorial	11 (73.3)	-	-
Lesion side, *n*%			
Left	3 (20)	-	-
Right	6 (40)	-	-
Both	6 (40)	-	-
NIHSS (for patients with stroke), mean ± SD	6.75 ± 3.42	-	-
Time from disease onset (months), mean ± SD	3.41 ± 1.36	-	-
Duration of tracheal intubation (months), mean ± SD	3.27 ± 1.62	-	-
Aspiration pneumonia, *n* (%)	13 (86.7)	-	-

Note: Gender was analyzed by Fisher’s exact test. Age, BMI, BI, and FOIS were analyzed by the two-independent sample *t*-test or Wilcoxon signed-rank test. BMI, body mass index; BI, Barthel index; FOIS, functional oral intake scale; NIHSS, National Institute of Health Stroke Scale.

## Data Availability

The data presented in this study are available on request from the corresponding author. The data are not publicly available due to privacy.
